# Updated estimation of the impact of a Japanese encephalitis immunization program with live, attenuated SA 14-14-2 vaccine in Nepal

**DOI:** 10.1371/journal.pntd.0005866

**Published:** 2017-09-21

**Authors:** Shyam Raj Upreti, Nicole P. Lindsey, Rajendra Bohara, Ganga Ram Choudhary, Sushil Shakya, Mukunda Gautam, Jagat Narain Giri, Marc Fischer, Susan L. Hills

**Affiliations:** 1 Child Health Division, Ministry of Health and Population, Kathmandu, Nepal; 2 Arboviral Diseases Branch, Centers for Disease Control and Prevention, Fort Collins, CO, United States of America; 3 Programme for Immunization Preventable Diseases, World Health Organization, Kathmandu, Nepal; Universidad Nacional Mayor de San Marcos, PERU

## Abstract

**Background:**

Japanese encephalitis (JE) is a mosquito-borne disease that is associated with considerable morbidity and mortality in many Asian countries. The objective of this study was to describe the impact of the JE immunization program using SA 14-14-2 JE vaccine implemented in Nepal during 2006 through 2011. A previous assessment after the initial program implementation phase described a significantly lower post-campaign JE incidence compared to expected incidence; however, the previous evaluation had limited post-campaign data for some districts.

**Methodology/Principal findings:**

JE and acute encephalitis syndrome (AES) data gathered through Nepal’s routine surveillance system from 2004 through 2014 were analyzed to assess the impact of the JE immunization program implemented in 31 districts. Expected incidence rates were determined by calculating the incidence of cases per 100,000 person-years in each district before the vaccination campaigns. This rate was applied to the relevant population after the vaccination campaigns, which provided the expected number of cases had the campaign not occurred. The observed incidence rate was the number of reported cases per 100,000 person-years post-campaign. Expected and observed JE and AES cases and incidence rates were compared. The post-campaign JE incidence rate of 0.7 cases per 100,000 was 78% (95% CI 76%–79%) lower than expected had no campaign occurred and an estimated 3,011 (95% CI 2,941–3,057) JE cases were prevented. The post-vaccination AES incidence of 5.5 cases per 100,000 was 59% (58%–60%) lower than the expected and an estimated 9,497 (95% CI 9,268–9,584) AES cases were prevented.

**Conclusions/Significance:**

This analysis strengthens previous findings of the substantial impact of Nepal’s JE immunization program using SA 14-14-2 JE vaccine.

## Author summary

In 2006, the Ministry of Health and Population in Nepal commenced a Japanese encephalitis (JE) immunization program using SA 14-14-2 JE vaccine, with mass campaigns conducted in selected districts, followed by introduction of JE vaccine into the routine childhood immunization program. JE and acute encephalitis syndrome data gathered through Nepal’s routine surveillance system from 2004 through 2014 were analyzed to assess the impact of this immunization program. Expected and observed JE and acute encephalitis syndrome cases and incidence rates were compared. Considerable impact on JE incidence was demonstrated and the results also suggested that a large proportion of acute encephalitis syndrome cases without laboratory confirmation are due to JE. The results support the belief that a JE immunization program will result in sizable reductions in the incidence of both laboratory-confirmed JE and clinical acute encephalitis syndrome cases. JE is a severe disease, and the program’s impact likely extended to reduction of rates of JE-related mortality and long-term disability.

## Introduction

Japanese encephalitis (JE) is a mosquito-borne disease that is associated with considerable morbidity and mortality in many Asian countries [1]. Approximately 20–30% of JE cases are fatal and 30–50% of survivors have neuropsychiatric sequelae [1,2]. There is no specific treatment for JE, but the disease is preventable by vaccination. During the past decade, there has been a substantial increase in the availability of improved JE vaccines, including the live-attenuated SA 14-14-2 JE vaccine [3]. The SA 14-14-2 vaccine has been found to be both safe and effective with over 96% protective effectiveness five years after administration of a single dose [4]. WHO recommends the use of JE vaccine in areas where JE is a public health priority, and has highlighted the need for good quality disease surveillance data to monitor vaccine impact [5].

JE was first recognized as a public health problem in Nepal in the mid-1970s [6,7]. In 2006, the Ministry of Health and Population (MOHP) in Nepal commenced an immunization program using SA 14-14-2 JE vaccine, and by 2009, the program had been implemented in 23 districts with the highest JE disease burden. An impact assessment in 2010 found that the post-campaign JE incidence rate was 72% lower than expected if no campaigns had occurred, and the clinical acute encephalitis syndrome (AES) incidence was 58% lower than expected [8]. However, limited post-introduction data in some districts might have impacted the accuracy of the assessment of the full impact of the program. In 2011, Nepal MOHP expanded the immunization program to an additional eight districts. By 2014, JE and AES surveillance data had been gathered for more than 3 years in all districts where the program had been implemented. In this assessment, we included JE and AES surveillance data from 2004 through 2014, thereby adding surveillance data for 2010–2014 for the 23 districts originally included in the impact analysis and data from 2004–2014 for the additional eight districts. We reviewed and analyzed these data to assess the impact the JE immunization program in the 31 districts.

## Methods

### Setting

Nepal is divided into three ecological zones from north to south; the Himalayan mountain region along the northern border with China, the hill region in the middle, and the Terai along the southern border with India. More than half of Nepal’s population of approximately 28 million live in the 24 districts of the Terai region. This area has conditions highly favorable for JE virus transmission; historically, more than 90% of JE cases in Nepal have been reported from this region [9,10]. In 1995, JE virus transmission was confirmed in Kathmandu Valley and endemicity was subsequently documented in other hill and mountain districts [11, 12]. In this analysis, as in the previous assessment, the campaign districts were categorized according to JE risk level [8]. Four western Terai districts (Kailali, Bardiya, Banke, and Dang) were classified as high risk; districts with lower incidence rates but recurrent seasonal JE virus transmission were classified as moderate risk.

### JE immunization program

From 2006 through 2011, mass immunization campaigns were conducted in 31 (41%) of Nepal’s 75 administrative districts, starting with Terai districts with the highest JE disease burden. By the end of 2011, campaigns had been implemented in all 24 Terai districts and seven (20%) of 35 hill districts. In 20 districts, campaigns targeted all persons aged ≥1 year; in the other 11 districts, only children aged 1–15 years were vaccinated. The median reported campaign coverage rate for the 31 districts was 90% (range: 59%–115%) [13]; coverage rates higher than 100% might have been due to residents of other districts being vaccinated and included in counts or underestimates of a district’s population. Routine JE immunization for children aged 12–23 months was introduced within 3 years of the mass campaign in each district. A single dose of SA 14-14-2 JE vaccine was used in campaigns and in the routine program.

### AES and JE surveillance in Nepal

Routine surveillance for AES cases in Nepal began in 1978. In 2004, AES and JE surveillance programs were strengthened, including the designation of 45 medical facilities as sentinel sites, initiation of enhanced case-based surveillance using a standardized case definition, and improved access to JE laboratory testing [10]. The initial 45 sentinel sites consisted of 34 sites in 20 Terai districts and 11 sites in 5 hill districts [8]. The AES/JE surveillance system was expanded to additional sites in subsequent years. AES cases identified at sentinel sites are reported to the Programme for Immunization Preventable Diseases at the World Health Organization (WHO) in Nepal. Cerebrospinal fluid (CSF) and serum specimens are collected when possible and tested at the National Public Health Laboratory or B.P. Koirala Institute of Health Sciences laboratory using a JE immunoglobulin (Ig) M antibody capture enzyme-linked immunosorbent assay (MAC-ELISA) [8]. Over the surveillance period, a variety of assays were used, including commercially available kits and laboratory-developed assays. Epidemiological and laboratory data were collected in a MOHP/WHO database maintained by WHO Nepal.

### Surveillance case definitions

Nepal uses WHO-recommended case definitions. An AES case is a person of any age, at any time of the year with the acute onset of fever and a change in mental status and/or new onset of seizures, excluding simple febrile seizures [14]. All cases that meet this definition are included in clinical AES surveillance, regardless of whether laboratory testing is performed. An AES case with JE virus-specific IgM antibody detected in CSF or serum is considered a JE case [14].

### Data analysis

JE and AES surveillance data for this analysis were included from the 45 reporting sites established in 2004, also used for the previous impact analysis, and from two sites established in hill districts in 2005. We included these two additional reporting sites because more than one third of AES cases from two of the new districts presented to these sites (and not to one of the 45 sites used in the previous analysis).

The same basic analysis methodology was used as documented in the previous assessment [8]. Briefly, JE and AES expected incidence rates were determined by calculating the incidence per 100,000 person-years in each district or age group before the vaccination campaign. This rate was applied to the relevant population after the vaccination campaign to calculate the expected number of cases had the campaign not occurred. The observed incidence rate was the number of reported cases per 100,000 person-years post-campaign. The differences between expected and observed incidence rates and exact binomial confidence intervals for the differences were calculated.

The cut-off dates between the “pre” and “post” vaccination campaign periods for each district were determined by selecting the mid-point of the vaccination campaign and adding 2 weeks to allow for development of immunity after vaccination. Campaigns were typically completed over a period of 2 weeks to 2 months. In three districts, partial campaigns were conducted in two consecutive years. To provide a conservative estimate of impact in these districts, the cut-off date definition was applied in the first year of the campaign. In short, cases that occurred from 2004 until 2 weeks following the mid-point of the vaccination campaign were used to calculate expected incidence rates, and those that occurred >2 weeks after the mid-point of the campaign until the end of 2014 were used to calculate observed rates.

The annual population data used for incidence calculations were obtained from the Nepal Department of Health Services’ Health Management Information System. These data are the official estimates used by the Nepal MOHP. SAS version 9.3 (SAS Institute, Cary, NC) and R version 3.1.2 were used for data analysis.

### Ethics statement

The protocol and analytical approach for this project were reviewed at the U.S. Centers for Disease Control and Prevention and the Nepal MOHP, respectively, and were considered to be program evaluation. Therefore, institutional review board review was not required. For epidemiological analysis, the data were anonymized.

## Results

### Impact on JE incidence and cases

**Fig 1:**
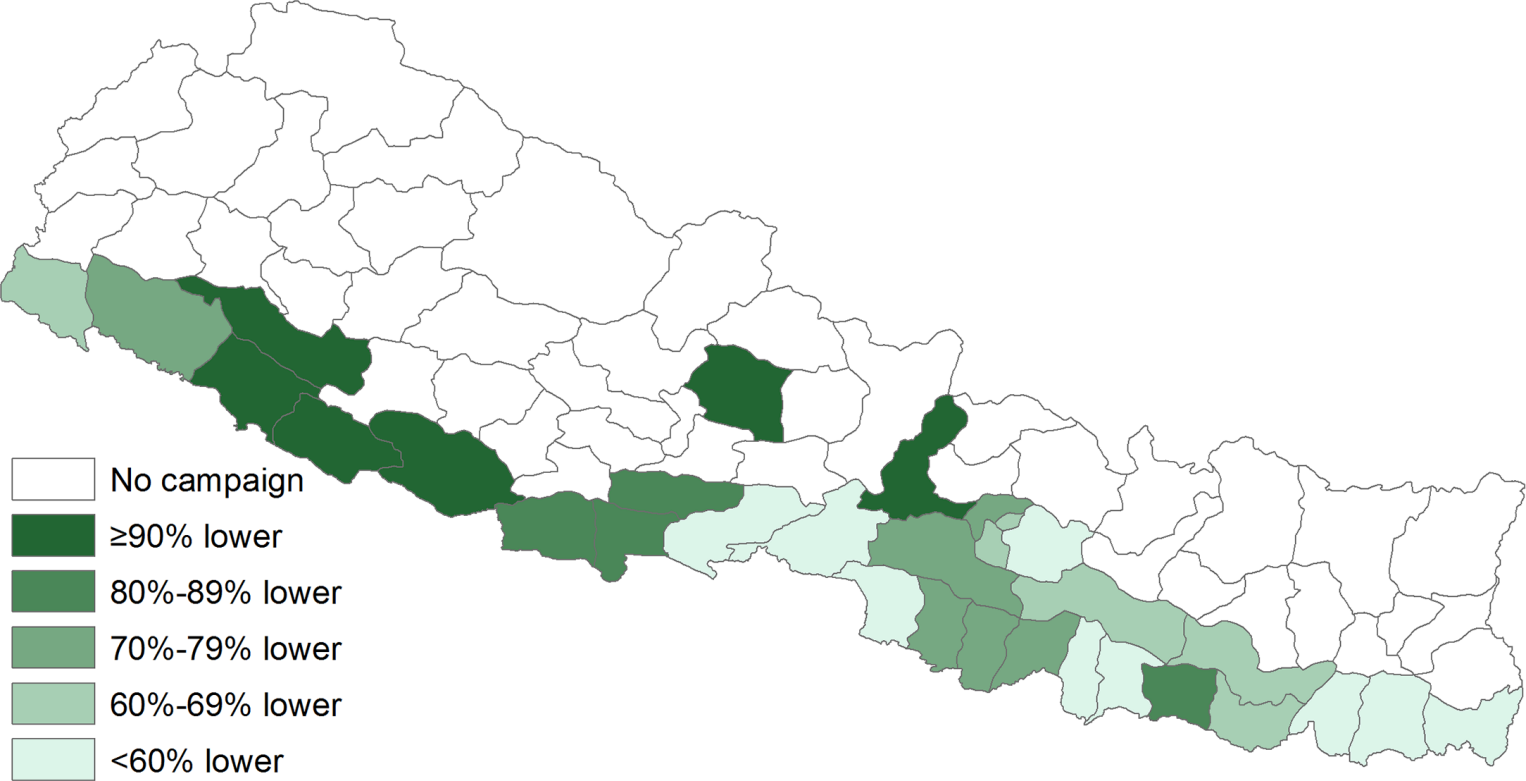
Percent difference in expected and observed incidence of Japanese encephalitis following vaccination campaign, by district, Nepal.

In the 31 districts, the observed post-campaign JE incidence rate was 0.7 cases per 100,000 person-years, 78% lower (95%CI 76%–79%) than the expected incidence of 3.3 cases per 100,000 (Table 1). The vaccination campaigns prevented an estimated 3,011 (95%CI 2,941–3,057) JE cases. The median difference between observed and expected incidence among the 31 districts was 71% lower (range: 100% lower–0% change) (Fig 1). The difference was significantly lower in 28 (90%) districts. The estimated impact was similar among all age groups (Table 2). The greatest impact was seen in the four high-risk Terai districts where the observed incidence rate was 89% lower (95%CI 87%–90%) than expected, and an estimated 1,955 JE cases were prevented. In the moderate risk areas, the observed post-vaccination incidence was 62% lower (95%CI 59%-65%) in the 20 Terai districts and 69% lower (95%CI 63%–75%) in the seven hill districts. An estimated 819 and 237 JE cases were prevented in those regions, respectively (Table 1).

**Table 1:** Japanese encephalitis expected and observed cases and incidence rates (IR) per 100,000 person years in Nepal.

	Campaign target population		Campaign year	Expected		Observed		Percent difference in IR	95% confidence interval
				Cases	IR	Cases	IR		
Vaccinated districts (n = 31)	Variable		2006–11	3,864	3.3	853	0.7	-78%	(-79%, -76%)
Ecologic and risk area									
Terai high-risk (n = 4)	≥1 yr		2006	2,202	11.7	247	1.3	-89%	(-90%, -87%)
Terai moderate risk (n = 20)	Variable		2006–11	1,319	1.6	500	0.6	-62%	(-65%, -59%)
Hill moderate risk (n = 7)		Variable	2008–11	343	1.9	106	0.6	-69%	(-75%, -63%)

**Table 2:** Japanese encephalitis expected and observed cases and incidence rates (IR) per 100,000 person years by age group in Nepal.

Age group	Expected		Observed		Percent difference in IR	95% confidence interval
	Cases	IR	Cases	IR		
<1	102	3.6	25	0.9	-75%	(-84%, -64%)
1–4	437	4.0	119	1.1	-73%	(-77%, -67%)
5–14	1,617	5.1	343	1.1	-79%	(-81%, -76%)
≥15	1,610	2.2	366	0.5	-77%	(-80%, -75%)

### Impact on AES incidence and cases

**Fig 2:**
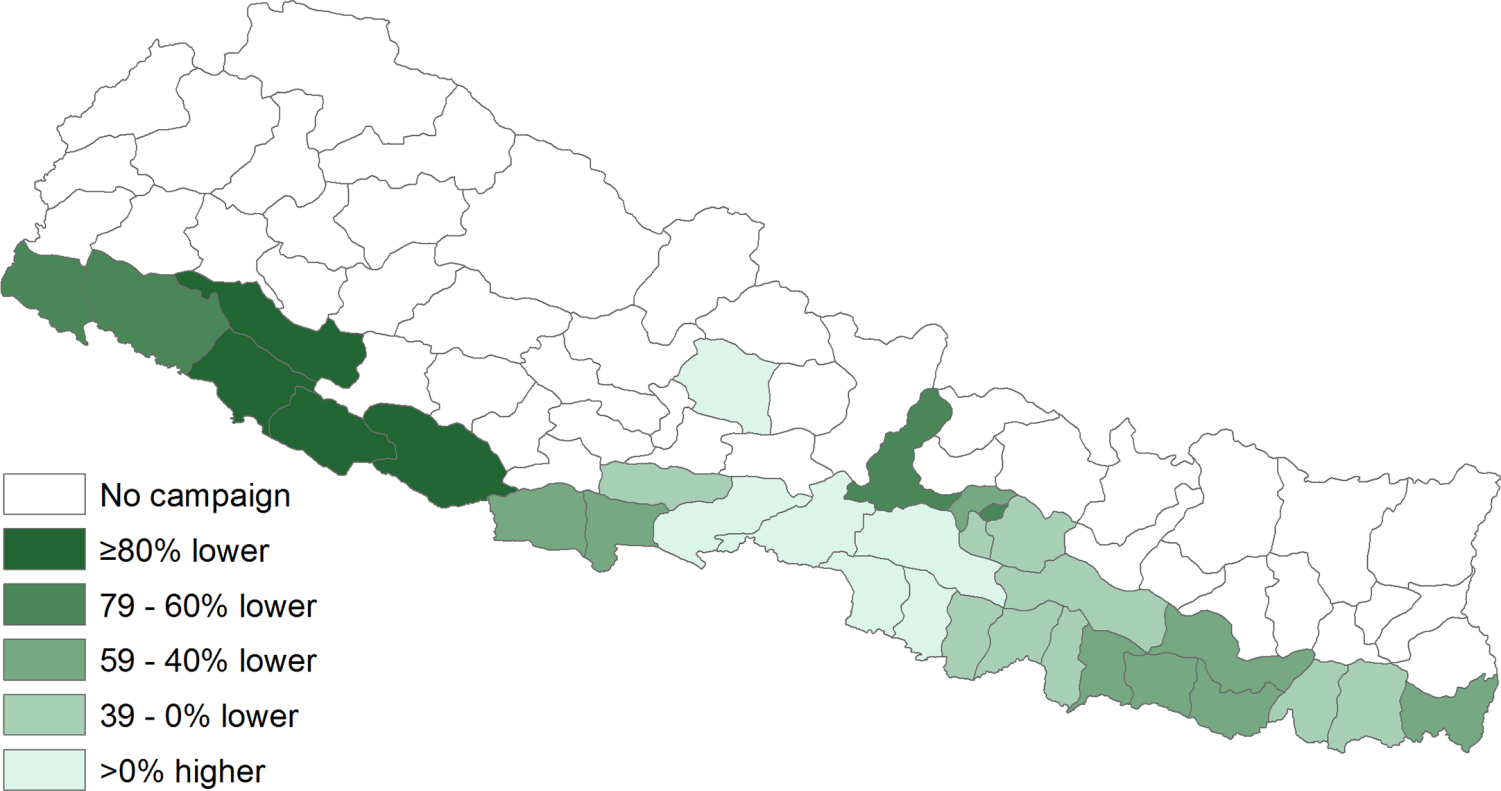
Percent difference in expected and observed incidence of acute encephalitis syndrome following Japanese encephalitis vaccination campaign, by district, Nepal.

The observed post-campaign AES incidence rate of 5.5 cases per 100,000 person-years was 59% lower (95%CI 58%–60%) than the expected incidence of 13.7 cases per 100,000 (Table 3). An estimated 9,497 (95%CI 9,268–9,584) AES cases were prevented by the vaccination campaigns. The median difference between observed and expected incidence was 41% lower (range: 96% lower–95% higher); the difference was significantly lower in 22 (71%) districts (Fig 2). Among the six districts where observed AES incidence was higher than expected, five were adjacent districts in the central Terai and one was in the central Hill region. As with JE, the greatest impact was in the four high-risk Terai districts where the observed incidence was 88% lower (95%CI 88%–89%) than expected. In the moderate risk areas, the observed incidence was 28% lower (95%CI 25%–30%) in the 20 Terai districts and 42% lower (95%CI 39%–45%) in the seven hill districts.

**Table 3:** Acute encephalitis syndrome expected and observed cases and incidence rates (IR) per 100,000 person years in Nepal.

	Campaign target population	Campaign year	Expected		Observed		Percent difference in IR	95% confidence interval
			Cases	IR	Cases	IR		
Vaccinated districts (n = 31)	Variable	2006–11	15,978	13.7	6,481	5.5	-59%	(-60%, -58%)
Ecologic and risk area								
Terai high-risk (n = 4)	≥1 yr	2006	7,830	41.5	905	4.8	-88%	(-89%, -88%)
Terai moderate risk (n = 20)	Variable	2006–11	5,897	7.4	4,267	5.3	-28%	(-30%, -25%)
Hill moderate risk (n = 7)	Variable	2008–11	2,251	12.6	1,309	7.3	-42%	(-45%, -39%)

## Discussion

This analysis strengthens previous findings on the substantial impact of Nepal’s JE immunization program using SA 14-14-2 JE vaccine. Following program implementation in 31 districts from 2006 through 2011, post-campaign JE incidence was 78% lower than expected and through 2014, >3,000 laboratory-confirmed cases of JE were estimated to have been prevented. Because several factors can prevent cases being laboratory-confirmed, including lack of sample collection, collection before IgM is detectable, or assay sensitivity issues, a greater proportion of AES might be caused by JE virus than suggested by laboratory-confirmed JE cases alone. With clinical AES incidence 59% lower than expected and almost 9,500 cases estimated to have been prevented, it suggests that the program’s impact on JE cases likely was much greater.

The prior impact assessment was conducted in the initial program implementation phase, when mass campaigns had been completed in 23 districts; it used surveillance data from 2004 through 2009. The analysis estimated that JE incidence was 72% lower than expected if no campaigns had occurred, and AES incidence was 58% lower [8]. Based on clinical AES cases prevented, the program’s impact was estimated to be about three times higher than suggested by prevention of laboratory-confirmed JE cases. The review period was relatively short and there were less than 6 months of post-campaign data in some districts at that time. This updated analysis, based on more than 3 years of post-campaign data in 31 districts, supports and strengthens the earlier findings.

As in the previous analysis, the largest impact of the program was seen in the four high-risk Terai districts. Awareness of the sizable disease risk and thus high vaccine uptake among the local population, and vaccination of everyone ≥1 year of age in the initial mass campaigns, likely contributed to this result. There are many possible reasons for the variability in the immunization program’s impact in other districts. These potentially could include programmatic factors (such as coverage rates), issues with vaccine handling affecting vaccine potency, and variability in surveillance over time. Data to investigate these possible factors were not available.

It was not surprising that the program’s impact was similar in all age groups. Children aged 1–14 years in all districts would have had the opportunity to receive JE vaccine through mass campaigns or, in post-campaign years, through the routine immunization program. All adults aged ≥15 years had an opportunity for vaccination during mass campaigns in 20 (65%) of the 31 districts. In the 11 remaining districts that targeted immunization to persons aged 1–15 years, campaigns were conducted in 2008 or prior; thus, young adults in those districts would also be protected in later years given the vaccine’s high long-term effectiveness [4]. Finally, mass campaigns have likely increased immunity in women of child-bearing age and young infants are probably protected by maternally-derived JE antibody, explaining the lower than expected post-campaign incidence rates in children aged <1 year.

JE incidence was significantly lower in 90% of districts (28/31) while AES incidence was significantly lower in 71% of districts (22/31). There are several possible reasons for this different impact on JE and AES incidence, and for the observed post-campaign AES incidence being higher than expected in six districts. There are many etiologies of AES in addition to JE that may influence AES patterns, including viral, bacterial, or parasitic infections [15, 16]. Monitoring AES surveillance data will generally be a good indicator of JE activity [17]. However, as this analysis indicates, monitoring laboratory-confirmed JE cases when possible is also important as using AES trends as a surrogate for JE trends could sometimes result in misleading information.

There were several limitations with this assessment. Temporal changes in the completeness and accuracy of the routine surveillance data might have occurred during the 11-year period, but this could not be assessed. The surveillance case definition did not change during the time period, but new hospitals were opened in some districts, and patterns of health-care seeking behavior may have changed. Additionally, the likelihood of an AES case being diagnosed with JEV infection was variable during the surveillance period because of changes in diagnostic testing practices; the percentage of AES cases with JE testing was ≤70% from 2004–2005, ≥94% from 2007–2009, and 88% from 2010–2014 [8]. Since testing generally increased following vaccine program implementation, this could have increased the number of JE cases reported over time without a true increase in disease incidence, resulting in an underestimate of the vaccine program’s impact. In addition, there were variations in the JE assays used over time. The accuracy of the annual population estimates used for incidence calculations also is unknown. In addition, it is not possible to determine the true level of JE virus transmission during 2004 through 2014. In some districts, including the four high-risk districts, pre-campaign incidence was based on surveillance data gathered for <3 years. In all districts, data used for the calculation of expected incidence included data from 2005, a high JE incidence year. Immunization likely prevented human JE outbreaks in subsequent years despite ongoing environmental transmission of JE virus. However, this analysis might have overestimated the true impact of the immunization program if JE virus activity was higher in districts in the pre-campaign years than in post-campaign years. Finally, vaccine impact can be affected by factors such as program coverage and vaccine efficacy, and reported coverage rates and vaccine potency after field distribution could not be confirmed.

Despite these limitations, this retrospective analysis using 11 years of surveillance data strengthens previous findings on the considerable impact of a JE immunization program with the SA 14-14-2 JE vaccine. The overall impact on JE cases was likely about three times higher than suggested by the laboratory-confirmed cases alone, supporting the idea that a JE immunization program will result in reductions in the incidence of both clinical AES cases and laboratory-confirmed JE cases. JE is a severe disease, and the program’s impact likely extended to reduction of rates of JE-related mortality and long-term disability. These findings support continued implementation of the JE immunization program in Nepal.

### Disclaimer

The findings and conclusions in this report are the authors and do not necessarily represent the official position of the Centers for Disease Control and Prevention.
